# Design of the Middle East African Registry for Women’s Cardiovascular Diseases: Protocol for a Multicenter Observational Study

**DOI:** 10.2196/72944

**Published:** 2026-07-30

**Authors:** Salma Charfeddine, Leila Abid, Sarra Chenik, Aiman Ghrab, Oussama Haddar, Iheb Ben Krayen, Selim Boudiche, Haithem Touati, Oumaima Ayedi, Mohamed Amine Ammar, Manel Ben Halima, Houssem Ben Ayed, Asma Brahim, Faten El Ayech, Emna Allouche, Houssem Thabet, Houaida Mahfoudhi, Taha Yassine Jabloun, Yasmine Ayadi, Alaeddine Ayadi, Ghassen Romdhani, Hassen Gargouri, Oumayma Zidi, Mohamed Ali Guedri, Rami Tlili, Bechir Trabelsi, Selim Hammami, Rim Othmen, Saoussen Antit, Syrine Saidane, Sirine Dardour, Skander Iddir, Elmahdi Kharrat, Anis Cheikhrouhou, Mohamed Derwich, Amal Mrabet, Taha Lassoued, Emna Rekik, Sahar Gmiha, Niez Laribi, Hakim Lamine, Zied Triki, Samir Ayari, Fatma Boujelbene, Essia Boughzela, Hajer Rekik, Ines Ben Ameur, Syrine Abid, Khalil Oueghlani, Abddayem Haggui, Afef Ben Halima, Wejdene Ouechtati, Emna Bennour, Rania Hammami, Mariem Jabeur, Ihsen Zairi, Mariem Drissa, Faouzi Addad, Sami Milouchi, Mohamed Sami Mourali, Hedi Ben Slima, Leila Bezdah, Elyes Neffati, Youssef Ben Ameur, Sondos Kraiem, Salem Kachboura, Ikram Kammoun, Lilia Zakhama, Hassen Ibn Hadj Amor, Khaldoun Ben Hamda, Yosra Messaoudi, Nejah Ben Hlima, Rana Dahmani, Habib Gamra, Zied Ibn Elhadj, Hichem Denguir, Chayma Ghorbel, Nizar Mechri, Samia Ernez Hajri, Alexandre Mebazaa, Fedi Ben Dhaou, Maroua Trigui, Wafa Fehri, Salem Abdesselem

**Affiliations:** 1 Cardiology Department, Hedi Chaker University Hospital, Route El Ain Km 0.5, Sfax, 3000, Tunisia, 216 98638435; 2 Cardiology Department, Military University Hospital, Tunis, Tunisia; 3 Cardiology Department, Habib Bourguiba University Hospital, Medenine, Tunisia; 4 Cardiology Department, La Rabta University Hospital, Tunis, Tunisia; 5 Cardiology Department, Menzel Bourguiba University Hospital, Bizerte, Tunisia; 6 Cardiology Department, Charles Nicolle University Hospital, Tunis, Tunisia; 7 Cardiology Department, Sahloul University Hospital, Sousse, Tunisia; 8 Cardiology Department, Mongi Slim La Marsa University Hospital, Tunis, Tunisia; 9 Cardiology Department, Habib Thameur University Hospital, Tunis, Tunisia; 10 Cardiology Department, Abderrahman Mami University Hospital, Ariana, Tunisia; 11 Cardiology Department, FSI La Marsa University Hospital, Tunis, Tunisia; 12 Private Cardiologist, Sfax, Tunisia; 13 Private Cardiologist, Tunis, Tunisia; 14 Cardiology Department, Djerba Hospital, Medenine, Tunisia; 15 Cardiology Department, Taher Sfar University Hospital, Mahdia, Tunisia; 16 Cardiology Department B, Fattouma Bourguiba University Hospital, Monastir, Tunisia; 17 Cardiology Department, Ibn El Jazzar University Hospital, Kairouan, Tunisia; 18 Cardiology Department, Military Hospital, Bizerte, Tunisia; 19 Cardiology Department A, Fattouma Bourguiba University Hospital, Monastir, Tunisia; 20 Cardiology Department, Taher Maamouri University Hospital, Nabeul, Tunisia; 21 Cardiology Department, Gabes Hospital, Gabes, Tunisia; 22 Cardiology Department, Kasserine Hospital, Kasserine, Tunisia; 23 Cardiology Department, Tatouine Hospital, Tatouine, Tunisia; 24 Cardiology Department, Farhat Hached University Hospital, Sousse, Tunisia; 25 Inserm MASCOT, AP-HP Department Of Anesthesia And Critical Care, Université Paris Cité, Hôpital Lariboisière, Paris, France; 26 Hygiene Department, Habib Bourguiba University Hospital, Sfax, Tunisia; 27 Cardiology Department, Clinic Pasteur, Tunis, Tunisia

**Keywords:** cardiovascular disease, registry, protocol, gender, heart failure, atrial fibrillation, ischemic heart disease, valvular heart disease

## Abstract

**Background:**

Cardiovascular disease (CVD) is a leading cause of morbidity and mortality in the Middle East and Africa (MEA), with a rising incidence particularly among women. Regional factors such as limited health care access, cultural barriers, and sex-specific risk factors exacerbate this burden. Despite this, women remain significantly underrepresented in cardiovascular research, and no large-scale, multicenter prospective trials have been conducted to provide national data. To address this gap, we established the Middle East African Registry Women Cardiovascular Disease (MEA-WCVD) to create a comprehensive database on the epidemiological profile and management of heart failure (HF), atrial fibrillation (AF), ischemic heart disease (IHD), and valvular heart disease (VHD) in women.

**Objective:**

The primary aim of this study is to compare the management of CVDs in women and men across MEA countries in accordance with current clinical practice guidelines. The study also seeks to identify gender-based disparities in health care insurance, income, and access to cardiovascular services.

**Methods:**

The MEA-WCVD is a prospective, multicenter, observational study enrolling consecutive patients aged ≥18 years with diagnosed HF, AF, IHD, or VHD across 25 tertiary care centers. Participants provide informed consent during a single visit, and trained investigators collect sociodemographic, clinical, and treatment data via electronic case report forms. The electronic case report form captures general characteristics (age, gender, and comorbidities) and diagnosis-specific details (imaging, guideline-based therapies, and complications). Data are stored in a centralized, contract research organization–managed database (Eshmoun-Clinical Research, Tunisia). An initial 75-day enrollment phase (May 2023-July 2023) is followed by a planned 1-year follow-up for outcome analysis. Data will be analyzed using SPSS (version 25) to compare gender disparities in management and outcomes using multivariable regression and survival analyses.

**Results:**

The MEA-WCVD study was funded in April 2023, and data collection began in May 2023. As of July 2023, a total of 15,366 participants have been enrolled across 25 centers. A 1-year follow-up is expected to be completed by July 2024. Data analysis is planned to commence in July 2024, with primary results anticipated for publication in March 2025. The study aims to establish the largest registry in the MEA region for HF, AF, IHD, and VHD, providing valuable insights into demographic trends, clinical management, and adherence to current guidelines.

**Conclusions:**

The MEA-WCVD registry will provide essential real-world data on the management and outcomes of the most prevalent CVDs (HF, AF, IHD, and VHD) in the MEA region. By directly comparing standard care management between men and women, this study will highlight gender disparities and inform future strategies for equitable cardiovascular care. The registry is expected to contribute to the largest contemporary cohort of patients with CVD in the region, advancing knowledge in cardiovascular epidemiology and clinical practice.

## Introduction

### Background

Numerous studies have uncovered substantial disparities in cardiovascular health between men and women, as well as among different subsets of women [1-3]. However, despite a growing global awareness, there remains a critical lack of region-specific data on the unique characteristics and management of cardiovascular diseases (CVDs) in women across the Middle East and Africa (MEA) region [4]. This research gap is particularly concerning as it contributes to the persistent underdiagnosis, undertreatment, and underresearch of common cardiovascular conditions such as ischemic heart disease (IHD), heart failure (HF), valvular heart disease (VHD), and atrial fibrillation (AF) in women.

Compounding this issue, several studies indicate that women with suspected heart disease are less likely than men to undergo recommended diagnostic testing and receive evidence-based treatments [3,5]. These disparities are further exacerbated by ongoing sex-based biases in clinical decision-making and the underrepresentation of women in cardiovascular research. While global research has increasingly acknowledged the influence of sex-specific pathophysiological, hormonal, and psychosocial factors in CVD, such insights are seldom translated into region-specific strategies, especially in low-resource settings.

In the MEA region, the challenges are magnified by limitations in health care infrastructure, sociocultural barriers, and insufficient awareness among both the public and health care professionals regarding the burden of CVD in women [1]. Moreover, existing prevention and treatment programs often fail to adequately address gender disparities, particularly in access to health care services, insurance coverage, and health education. These shortcomings are increasingly alarming given the demographic shift toward an aging population and the rising prevalence of cardiovascular risk factors in the region. This evolving epidemiologic landscape suggests a significant surge in CVD-related morbidity and mortality in the near future, with profound implications for public health systems.

In recent decades, Africa has seen a notable shift in the patterns of CVD, marked by a decline in rheumatic heart disease and a rising prevalence of noncommunicable conditions such as AF, HF, and IHD. While some national registries—such as those in Tunisia—have shed light on these trends [6,7], there remains a dearth of large-scale, gender-focused cardiovascular registries encompassing the broader MEA region. Notably, no comprehensive registry to date has centered on the cardiovascular health of women in this context, highlighting an urgent need for sex-disaggregated data to inform health policy and clinical practice.

### Research Gap

There is a lack of robust, sex-disaggregated, and region-specific data on the clinical presentation, management, and outcomes of CVDs in women within the MEA region. Existing studies and registries have largely overlooked gender-based analysis and fail to account for socioeconomic and structural determinants of health.

### Registry Objectives

The primary goal of the Middle East African Registry Women Cardiovascular Disease (MEA-WCVD) is to compare the diagnosis, treatment, and outcomes of major CVDs in women and men across participating MEA countries in alignment with current clinical practice guidelines.

The secondary objectives are to identify gender-based disparities in health care access, including differences in insurance coverage, income level, and health care use, as well as highlight any deviations from guideline-recommended care based on sex.

### Hypothesis

We hypothesize that women in the MEA region with CVDs are less likely than men to receive appropriate diagnostic evaluation and evidence-based treatment, resulting in suboptimal clinical outcomes. Furthermore, we expect that socioeconomic factors such as limited health care coverage and reduced access to specialized services disproportionately affect women’s cardiovascular care.

## Methods

### Study Design

The MEA-WCVD is a prospective, observational, multicentric international study. This publication will focus on the available Tunisian cohort across all governorates of Tunisia, representing patients from all governorates across the public and private health care sectors.

The study aims to enroll at least 2000 female patients from Tunisia, with the participation of hospital and private cardiologists who agree to the study protocol.

**Oversight and Leadership**

The protocol for the MEA-WCVD was approved by the Tunisian Society of Cardiology and Cardiovascular Surgery and coordinated by a team headed by 2 expert female cardiologists.

### Inclusion and Exclusion Criteria

Patients (men and women) are officially enrolled in the MEA-WCVD study only if they are aged >18 years and have at least 1 of the following 4 diagnoses: HF, AF, IHD, or VHD.

Individuals are excluded if they present with CVDs other than the 4 target conditions. Additionally, patients who are unable to provide informed consent—due to cognitive impairment, communication barriers, or other medical or legal reasons—or those who explicitly refuse to participate in the study are also excluded.

### Clinical Assessment and Data Collection

During the office visit, the physician completes the electronic case report form (eCRF) of the registry after the patient’s approval according to the inclusion criteria.

To ensure the accuracy and completeness of the data, all centers involved in data collection adhere to standardized protocols for data entry, with regular training sessions provided to research personnel.

The eCRF is structured into 2 main sections. The first is a common data module that includes a comprehensive set of items pertaining to each patient’s sociodemographic profile and general clinical background. This encompasses variables such as age, sex, level of education, type of health insurance, socioeconomic status, degree of financial or physical dependence, and smoking status. Additionally, it records information on cardiovascular risk factors (eg, hypertension, diabetes, or dyslipidemia), past cerebro-cardiovascular events (such as stroke or myocardial infarction), coexisting pulmonary diseases, thyroid disorders (hypothyroidism or hyperthyroidism), and any history of cancer and related treatments. Key laboratory data such as creatinine and hemoglobin levels are also collected to assess renal function and anemia status, both of which are important prognostic factors in cardiovascular care.

The second part of the eCRF is diagnosis specific and tailored according to the patient’s primary inclusion diagnosis (HF, AF, IHD, or VHD). For each condition, this section captures detailed clinical information, including the etiology, findings from relevant imaging and laboratory tests, the use of guideline-directed medical therapy, occurrence of complications, and aspects related to medication adherence and reported side effects. This diagnosis-specific approach allows for a nuanced evaluation of each disease entity and facilitates meaningful comparisons between male and female patients across the MEA region.

The study began with an initial 75-day enrollment phase, which took place from May 2023 to July 2023. During this phase, eligible participants were identified and enrolled in the study. After the enrollment phase, a planned 1-year follow-up period will be conducted to analyze the outcomes associated with the treatment. This follow-up will be carried out through patient visits to health care centers. The 1-year follow-up period will allow for the evaluation of both short- and long-term treatment effects, contributing to a more comprehensive understanding of the study’s outcomes. The protocol for the MEA-WCVD was approved by the Tunisian Society of Cardiology and Cardiovascular Surgery and coordinated by a team headed by 2 expert female cardiologists.

### Ethical Considerations

This study is conducted in compliance with the principles outlined in the Declaration of Helsinki. The MEA-WCVD (ClinicalTrials.gov ID: NCT05869214) received ethics approval from the South Tunisian Persons’ Protection Committee (0496/2023). The protocol was reviewed and approved as a multicenter study involving both hospital-based and private practice cardiology consultations.

All eligible participants are enrolled only after providing written informed consent during a single outpatient cardiology visit. Investigators ensure that participants fully understand the objectives of the registry, the voluntary nature of their participation, and their right to withdraw at any time without any impact on their medical care.

Data collected via the eCRF include anonymized and pseudonymized identifiers. No identifiable personal data (eg, names, addresses, or national IDs) are stored in the analysis database. All data are securely transferred and stored in a centralized, certified, and encrypted database managed by the contract research organization (Eshmoun, Tunisia).

No financial or material compensation is provided to participants. Participation in the study is voluntary, and no incentives or reimbursements are offered to avoid coercion or bias.

No identifiable photographs, images, or multimedia files of participants are included in this manuscript.

### Statistics

Data will be analyzed using SPSS (version 25; IBM Corp). To explore gender disparities in cardiovascular health outcomes and treatment effectiveness, data will be stratified by gender during analysis.

For the descriptive analyses, the Kolmogorov-Smirnov normality test will be performed for quantitative variables. Means and SDs will be used to describe quantitative variables when they are normally distributed. Otherwise, medians and IQRs will be used. Qualitative variables will be presented as numbers and proportions.

To compare frequencies based on gender, the Pearson chi-square test will be used when the application conditions are verified. Otherwise, the Fisher exact test will be used. To compare means based on gender, the Student 2-tailed *t* test for independent samples will be used when the 2 quantitative variables are normally distributed. If at least one variable is not normally distributed, the Mann-Whitney *U* test will be used. *P* values of less than .05 will be considered statistically significant.

## Results

The MEA-WCVD study was officially funded in April 2023, with data collection commencing in May 2023. By July 2023, a total of 15,366 participants had been enrolled across 25 centers. The study plans to complete a 1-year follow-up by July 2024, after which data analysis will begin. The primary results are expected to be published in March 2025.

This study seeks to establish the largest registry in the MEA region for HF, AF, IHD, and VHD. By examining these conditions, the study aims to provide valuable insights into regional demographic trends, clinical management practices, and patient adherence to current medical guidelines. The findings will be pivotal in shaping future health care strategies and improving the management of CVDs in the region.

## Discussion

This registry is expected to reveal significant sex-based disparities in the management and outcomes of CVDs among patients in MEA countries. Thus, the MEA-WCVD will fill a critical data gap by offering region-specific, sex-disaggregated insights into 4 major cardiovascular conditions: HF, AF, IHD, and VHD [4].

### Anticipated Findings

The primary analyses are expected to confirm disparities in cardiovascular care and outcomes between men and women, particularly in diagnostic approaches, therapeutic interventions, and access to care. For instance, we hypothesize that fewer women will undergo advanced imaging or invasive procedures or receive guideline-directed medical therapy compared to men. Furthermore, socioeconomic and health care access factors—such as insurance status and financial dependence—may further exacerbate these disparities in women. This comprehensive analysis will offer a deeper understanding of the structural and systemic barriers affecting cardiovascular care for women in the MEA region.

### Comparison to Prior Work

While previous registries and studies conducted in high-income countries have shown persistent sex-based differences in cardiovascular health, such investigations remain scarce in the MEA region [1-3]. Existing studies from North Africa, such as those from Tunisia, have primarily focused on single diseases and lack large-scale, sex-disaggregated data. The MEA-WCVD is unique in its multicentric, multisite approach and its focus on capturing real-world clinical practices in both men and women across a wide range of health care settings [6,7].

### Strengths and Limitations

A major strength of this study lies in its prospective design and standardized electronic data collection, which enhances the accuracy and consistency of the data across sites. In addition, the focus on sex-disaggregated outcomes and the inclusion of social determinants of health will allow for a multidimensional understanding of gender disparities in cardiovascular care.

However, limitations may include potential selection bias, differences in health care infrastructure between participating countries, and challenges related to the availability and interpretation of diagnostic tests across diverse settings. Although this study provides valuable insights based on a 1-year follow-up, extending the follow-up period in future research will be essential to better capture the long-term effects of cardiovascular treatments, monitor chronic disease progression, and assess the sustainability of treatment outcomes, particularly among women.

The inclusion of genetic data or biomarkers could have further enhanced our ability to identify populations at higher risk and support the development of personalized treatment strategies. However, the integration of genetic or molecular data presents significant challenges, including logistical and financial constraints, which prevented their inclusion in this analysis.

### Future Directions

The MEA-WCVD has the potential to inform future health policies, educational initiatives, and targeted interventions aimed at reducing gender inequities in cardiovascular care. The findings may also serve as a foundation for the development of regional clinical guidelines that better reflect the realities of health care delivery in MEA countries. Additional longitudinal follow-up could be considered to assess the impact of interventions over time and explore outcomes such as rehospitalization and mortality.

### Dissemination Plan

All collected data are pseudonymized to ensure patient confidentiality and will be analyzed centrally before being shared in aggregate form. The results of the study will be compiled into a comprehensive report and disseminated through peer-reviewed publications and conference presentations. Importantly, participating investigators will have access to the final analyses, and efforts will be made to share the findings with local health authorities, medical societies, and advocacy groups to support evidence-based decision-making and gender-sensitive health care planning.

### Conclusions

The MEA-WCVD represents a major step toward addressing the significant lack of sex-specific cardiovascular data in MEA countries. By providing real-world, region-specific evidence on the management and outcomes of AF, HF, IHD, and VHD, this study is expected to uncover critical disparities in care between women and men. The anticipated findings will not only contribute to a better understanding of the epidemiologic profile of CVD in the MEA region but also help inform more equitable and guideline-oriented clinical practice. Ultimately, this large-scale registry has the potential to guide policy, optimize resource allocation, and foster gender-sensitive improvements in cardiovascular health systems across the region.

## Abbreviations

AF: atrial fibrillation
CVD: cardiovascular disease
eCRF: electronic case report form
HF: heart failure
IHD: ischemic heart disease
MEA: Middle East and Africa
MEA-WCVD: Middle East African Registry Women Cardiovascular Disease
VHD: valvular heart disease
